# Adjustable dual-balloon therapy: quality of life after prostate incontinence treatment

**DOI:** 10.1007/s11255-025-04945-w

**Published:** 2025-12-04

**Authors:** Daniela Hinchman-Dominguez, Julie Klock, Michael P. Feloney, Sankalp Vinayak

**Affiliations:** 1 Department of Urology, Creighton University School of Medicine, 7710 Mercy Road, Omaha, NE 68124 USA; 2 Present Address: Buffalo General Medical Center, 100 High Street, Buffalo, NY 14203 USA; 3 Present Address: Cleveland Clinic, 9500 Euclid Avenue, Cleveland, OH 44195 USA

**Keywords:** Urinary incontinence, Prostatectomy, Urinary sphincter, Artifical, Quality of life, Postoperative complications, Treatment outcome

## Abstract

**Purpose:**

To assess the quality of life (QoL) and surgical outcomes of adjustable dual-balloon continence therapy (DBACT), ProACT™ is a minimally invasive treatment for post-prostatectomy urinary incontinence.

**Methods:**

In this retrospective, single-surgeon review conducted between September 2020 and March 2023, 21 patients with moderate-to-severe post-prostatectomy incontinence underwent ProACT™ placement at a Midwest academic center. A chart review was conducted to extract procedural and outcomes data. Postoperative QoL surveys, including pads-per-day (PPD) and incontinence QoL (IQoL) scores, were administered.

**Results:**

The average patient age was 70 years. Six (28.6%) of the 21 patients had prior radiation. The average operating time was 61.14 min. Patients needed an average of 3.33 adjustments post-surgery. The average decrease in PPD was 5.93, with 81% of patients achieving meaningful dryness with the use of ≤ 2 PPD. 76% of patients felt that ProACT™ helped their incontinence, improved their QoL, was worthwhile, and would recommend it to others. Of the 15 non-radiated patients, one had complications (6.67%). Of the 6 radiated patients, 5 (83.3%) had complications. There were no erosions in patients who were not previously radiated. There were no serious complications requiring prolonged hospitalization, such as sepsis or fistula formation.

**Conclusions:**

ProACT™ is a safe and effective treatment for incontinence after prostatectomy with high patient satisfaction and a low complication rate in patients without prior radiation. For radiated patients, ProACT™ can be efficacious, but they should be counseled about slower improvement times and higher complication rates.

**Supplementary Information:**

The online version contains supplementary material available at 10.1007/s11255-025-04945-w.

## Introduction

Urinary incontinence is a distressing condition that affects many urology patients. Although common, urinary incontinence can be difficult to treat, and its impact is multifaceted and often not openly discussed. Beyond treatment costs, urinary incontinence disrupts activities of daily living, quality of life, confidence, and self-image.

There are three types of urinary incontinence: stress, urge, and overflow [1]. A commonly accepted fourth category is ‘mixed’, which involves more than one type of urinary incontinence. Men who undergo radical prostatectomy (RP) and develop urinary incontinence most commonly have stress urinary incontinence (SUI) [2]. Even with experienced surgeons using minimally invasive techniques, approximately 1 in 5 men undergoing prostatectomy experience SUI postoperatively [3, 4]. A recent prospective non-randomized trial including 2625 men evaluated surgical approaches for RP and found men experienced similar rates of urinary incontinence at 20.2% and 21.3% for open retropubic RP and robotic-assisted RP, respectively [5]. Although most cases of post-prostatectomy urinary incontinence (PPUI) resolve within a year of surgery, many men continue to have PPUI after a year despite treatment with conservative therapy, with estimates ranging from 4% to 69%, depending on the definition used [6].

There are currently several hypotheses proposed describing the mechanism for PPUI, most of which describe damage or injury to structures necessary for maintaining continence, such as the internal sphincter and external rhabdosphincters with resulting intrinsic sphincter deficiency. Other possible etiologies include disruptions to the neurovascular bundle and partial denervation of the bladder incurred because of radiotherapy in patients requiring adjuvant radiation therapy or secondary to extensive dissection during surgery in RP [2]. There is some evidence that better rates of continence post-RP can be achieved using specific surgical techniques such as bladder neck-sparing, Retzius-sparing, or the Bocciardi approach, high nerve release, and fixation of the bladder–urethra anastomosis using a Rocco stitch, but the strongest evidence supports the bilateral nerve-sparing approach for preserving continence and erectile function when oncologically feasible [7]. Beyond preoperative bladder function and intraoperative surgical techniques, advanced age, obesity, prior prostate cancer treatment or instrumentation, higher tumor grade group, and higher prostate-specific antigen levels are preoperative risk factors that are reported to increase the likelihood of developing PPUI and should be accounted for by the operating surgeon [4].

For patients who still experience symptoms of PPUI, first-line management consists of pelvic floor muscle training (PFMT) and lifestyle adjustments. These adjustments include modifying diet or fluid intake, reducing caffeine intake, and increasing physical activity when possible. For patients with persistent incontinence beyond 12 months in the postoperative period, treatment with behavioral management and external devices and/or surgical management is recommended, per the American Urologic Association (AUA) Guidelines. Therapeutic decisions should be made concordantly with the surgeon and patient to best account for clinical and social factors that may impact treatment success.

As of 2024, the AUA guidelines conditionally recommend “adjustable balloon devices to non-radiated patients with mild to severe stress urinary incontinence after prostate treatment.” with an evidence level of Grade C. Artificial urinary sphincters (AUS) and male slings are also recommended for patients’ PPUI, with strong and moderate recommendations respectively and with an evidence level of Grade B for both [8]. The surgical options for PPUI are tiered according to best fit, patient’s clinical history, and needs. Other factors, including age, concomitant medical conditions, and severity of incontinence, need to be considered.

**Fig. 1:**
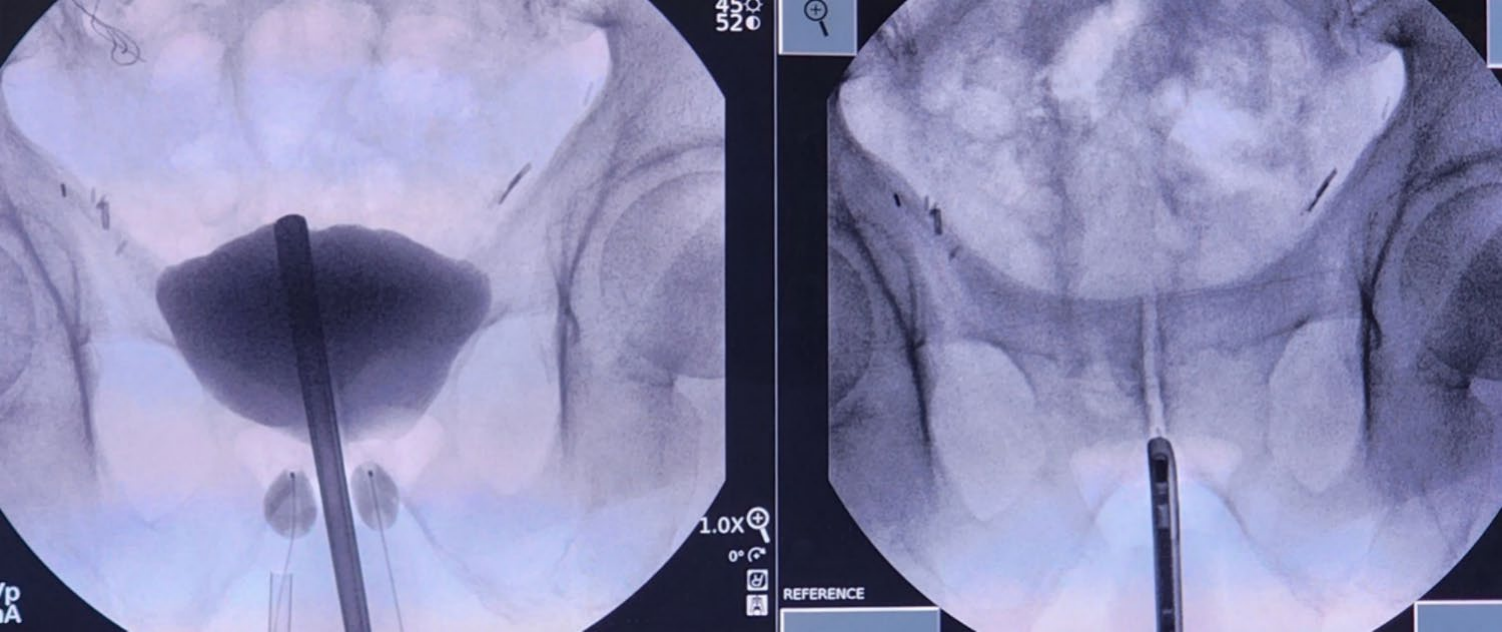
Left: Appropriate placement of DBACT device. Right: Anatomic reference of cystoscope at bladder neck. Used with patient and physician permission

**Fig. 2:**
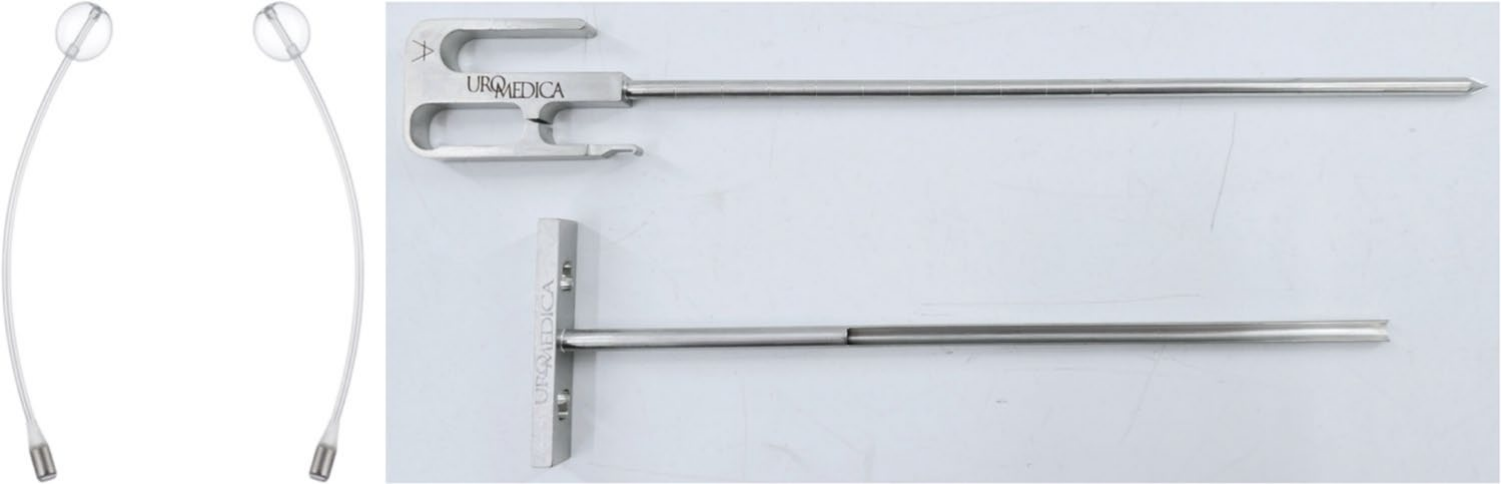
Left: Pair of ProACT balloons. Right: Trocar used to implant balloons

**Fig. 3:**
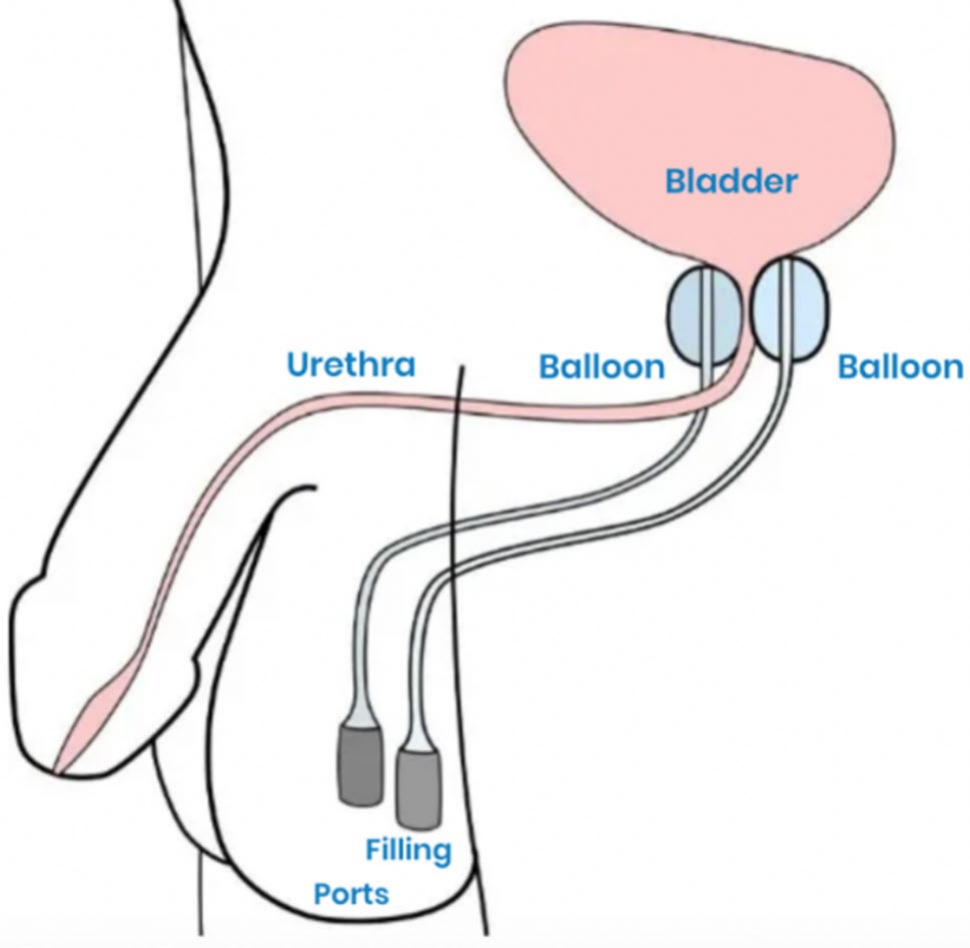
Schematic of DBACT. Used with permission from UroMedica

While the AUS is considered the ‘gold standard’ treatment, it may not be suitable for every patient, notably those lacking manual dexterity, those who are poor surgical candidates, and those who would prefer trying minimally invasive therapies in a more graded approach. In the United States, one emerging therapy for these patients is the dual-balloon adjustable continence therapy (DBACT). The DBACT consists of 2 silicone balloon devices, which are placed above the urogenital diaphragm on either side of the urethra, below the bladder neck, increasing bladder outflow resistance and improving continence (Fig. 1). The periurethral silicone balloons are connected to titanium ports via a silicone conduit (Fig. 2). The ports are tunneled into the scrotum, which allows them to be easily accessible percutaneously for in-office adjustments via a 23-gauge needle (Fig. 3).

The DBACT is an attractive alternative to AUS, offering patients a less-invasive, quicker procedure with no need for manipulation of the device in order to void. Unlike the AUS, the DBACT is adjustable postoperatively, which can improve incontinence after initial implantation, allowing for optimization of continence over time.

DBACT was approved by the FDA in 2015. In the United States, DBACT is sold as the ProACT™ device, manufactured by Uromedica Inc., Plymouth, MN, USA. In the European Union, ProACT and similar dual-balloon systems have been used since 2002 with long-term efficacy. A 2023 meta-analysis of 18 studies showed 55.2% of patients achieving dryness, as defined as 0–1 pads per day (PPD), post-operatively and 67.5% patient satisfaction [9]. While well-established, outcomes remain heterogeneous, and the device is best regarded as an established alternative rather than a universal standard.

In this study, we investigated the efficacy, patient satisfaction, and safety of DBACT in the treatment of PPUI.

## Materials and methods

This was a retrospective, single-surgeon, single-center study evaluating consecutive patients who underwent ProACT implantation for post-prostatectomy incontinence. Inclusion criteria included men with moderate-to-severe stress urinary incontinence for > 12 months after prostatectomy despite conservative management. Exclusion criteria included prior urethral stricture, active infection, or unresolved malignancy. We evaluated 21 patients who underwent DBACT as surgical treatment of PPUI between September 2020 and March 2023 at a tertiary academic medical center in the American Midwest. Preoperative assessment included medical and surgical history, along with surveys regarding urinary continence (Supplementary Fig. 1). Daily pad count was used to quantify the severity of urinary incontinence, classified as mild (1–2 pads), moderate (3–4 pads), or severe (≥ 5 pads). From retrospective chart review, the number of PPD reported by patients pre- and post-operatively, the number of adjustments needed, volume of adjustments made to decrease volume (mL), volume of adjustments made to increase volume (mL), initial volume (mL), ultimate volume (mL), length of procedure (minutes), blood loss (mL), prior radiation, previous procedures undergone to treat UI, complications, and the number of revisions were recorded.

All patients were administered an Incontinence Quality of Life (IQoL) survey at follow-up. This IQoL survey was adapted from the Male Urinary Symptom Impact Questionnaire (MUSIQ) with an abbreviated list of questions in regard to continence and activity of daily living with additional questions specific to satisfaction with the ProACT device, including whether the patient found the surgery worthwhile and whether they would have the surgery again [10]. IQoL surveys were administered on paper in person, by phone, and by mail. IQoL survey responses were graded on a Likert scale from 0 (“Strongly Disagree”) to 3 (“Strongly Agree”). Results were recorded in a secure database.

Postoperative survey data was gathered and represented in percentages. PPD was compared pre- and post-operatively, and the percentage change in PPD was calculated. Given the small sample size, a Mann–Whitney *U* test was used to compare changes in PPD between the radiated and non-radiated groups. Because of the small sample size, Fischer’s Exact tests were used to compare complication rates, social dryness, and recommendation of the device between the radiated and non-radiated groups. All patients who underwent device implantation were included in the data analysis, including patients who later had the device explanted.

## Results

### Patient characteristics

Patient age ranged from 57 to 81 years, with the average age being 70.18 years. Six patients (28.6%) had undergone radiation therapy (RT) prior to their DBACT procedure. On average, patients used 7.81 PPD at baseline (SD = 5.94) (Table 1). Previous therapies and procedures patients failed prior to DBACT implantation included penile clamp, condom catheter, male sling, and AUS, with 7 (33.3%) patients being prior intervention naïve. Nineteen (90.5%) patients completed at least 12 months of postoperative follow-up evaluation. The remaining two (9.5%) patients had completed at least 4 months of postoperative follow-up. The average follow-up time was 20.95 months [standard deviation (SD) = 10.43].

**Table 1:** Patient characteristics per patient medical records presented as a number or average

Characteristics	Total (*n* = 21)	Radiated (*n* = 6)	Non-radiated (*n* = 15)
Sex (M)	21	6	15
Age, years	70.18 (SD = 6.95)	70.67 (SD = 6.83)	70.00 (SD = 7.22)
Prior interventions			
Penile clamp	9	5	4
Condom catheter	3	2	1
Male sling	2	0	2
AUS	3	0	3
Radiated	6	6	0
Pads per day pre-op	7.81 (SD = 5.94)	9.83 (SD = 5.61)	7.00 (SD = 6.04)

### Operative characteristics

For the initial DBACT implant, the average operating time was 61.14 min (SD = 17.23). An average blood loss of 3.20 mL (SD = 2.20) was observed. No significant bleeding or anesthetic complications were seen in any of the patients. Patients left the operating room with an initial balloon volume of 1.0 or 1.5 mL. Patients, on average, needed 3.33 adjustments postoperatively (SD = 2.24), with adjustments occurring a minimum of 1 month apart. An average total additional volume of 2.57 mL (SD = 2.02) saline was added to initial balloon volumes over several months at a time.

### PPD reduction

According to postoperative survey data, 17 (81%) patients achieved meaningful dryness with the use of ≤ 2 PPD, 11 (52.3%) patients of which were socially dry with the use of ≤ 1 PPD reported on the questionnaire. There was a significant difference in social dryness, with none of the radiated patients (0 of 6, 0%) achieving social dryness, compared to 11 of 15 (73.3%) non-radiated patients (odds ratio (OR) 0.0, *p* = 0.0039; Fisher’s exact test). The average decrease in PPD from all patients was 5.93 pads (SD = 5.72). The average reduction in PPD in the radiated group was 6.0 pads (SD = 5.50). The average reduction in PPD in the non-radiated group was 5.90 pads (SD = 5.98) (Table 2). Data exhibited high variability (SD ≈ mean), suggesting non-normal distribution of continence outcomes. There was no statistically significant difference in PPD reduction in radiated and non-radiated patients (*U* statistic = 43.5, *p* = 0.94; Mann–Whitney *U* test), with the median change being 3.5 PPD vs. 4.0 PPD, respectively.

**Table 2:** Post operative survey data

	Total (*n* = 21)	Radiated (*n* = 6)	Non-radiated (*n* = 15)
Pads per day post-op, *n* (%)			
Socially dry (0–1 PPD)	11 (52.3%)	0 (0%)	11 (73.3%)
2-3PPD	6 (28.6%)	2 (33.3%)	4 (36.4%)
4-5PPD	4 (19.0%)	4 (66.7%)	0 (0%)
Average change in PPD	5.93 (SD = 5.72)	6.0 (SD = 5.50)	5.90 (SD = 5.98)
Average percent change in PPD, %	72.19% (SD = 23.74)	53.61% (SD = 20.99)	79.62% (SD = 21.00)
Improvement in QoL, *n* (%)			
Strongly agree	12 (57.1%)	4 (66.7%)	8 (53.3%)
Agree	5 (23.8%)	0 (0%)	5 (33.3%)
Disagree	2 (9.5%)	2 (33.3%)	0 (0%)
Strongly disagree	2 (9.5%)	0 (0%)	2 (13.3%)
DBACT helped, *n* (%)			
Strongly agree	13 (62.0%)	3 (50.0%)	10 (66.7%)
Agree	4 (19.0%)	1 (16.7%)	3 (20.0%)
Disagree	3 (14.3%)	2 (33.3%)	1 (6.7%)
Strongly disagree	1 (4.7%)	0 (0%)	1 (6.7%)
DBACT Worthwhile, *n* (%)			
Strongly agree	13 (62.0%)	3 (50.0%)	10 (66.7%)
Agree	3 (14.3%)	0 (0%)	3 (20.0%)
Disagree	3 (14.3%)	3 (50.0%)	0 (0%)
Strongly disagree	2 (9.5%)	0 (0%)	2 (13.3%)
Recommend DBACT to someone else? *n* (%)			
Yes	17 (81.0%)	4 (66.7%)	13 (86.7%)
No	4 (19.0%)	2 (33.3%)	2 (13.3%)

The outcome on the x-item questionnaire is presented as a number (%) or average

### QoL improvements

On the postoperative IQoL survey, Patients reported high satisfaction with life (mean = 2.33, SD = 0.73), health (mean = 2.29, SD = 0.64), and urinary incontinence (mean = 2.10, SD = 1.09). Improvements in daily functioning related to urinary symptoms were also noted across multiple domains, including household chores (mean = 1.62, SD = 1.24), travel (mean = 1.80, SD = 1.10), social life (mean = 1.86, SD = 1.20), emotional well-being (mean = 1.62, SD = 1.33), physical well-being (mean = 1.67, SD = 1.28), and ability to participate in entertainment (mean = 1.52, SD = 1.25) (Table 2).

Overall, 81.0% (17/21) agreed that the device improved their continence, 76.2% (16/21) felt it was worthwhile, and 81.0% (17/21) would recommend the procedure. Among non-radiated patients (*n* = 15), agreement was high: 86.7% agreed the device improved continence, 93.3% felt it improved QoL, 86.7% said it was worthwhile, and 86.7% would recommend it. Among radiated patients (*n* = 6), 66.7% agreed the device helped continence, QoL, and was worth it; 66.7% would also recommend it. There was no significant difference in the proportion of patients who themselves would recommend the ProACT device between radiated (4 of 6, 66.7%) and non-radiated (13 of 15, 86.7%) patients (OR 0.31, *p* = 0.544; Fisher’s exact test).

### Operative complications

15 (71.4%) patients experienced no complications. Complications necessitating revision included inability to adjust left balloon due to equipment malfunction (*n* = 1), bladder perforation (*n* = 2), urethral erosion (*n* = 1), and traumatic urethral perforation (*n* = 1). Of the 6 patients who experienced one of the complications described, 5 (83.3%) had undergone prior RT. Complication rates were significantly higher among radiated patients compared to non-radiated patients (OR 70.0, *p* = 0.0017; Fisher’s exact test). Only one patient who did not have prior radiation had an operative complication (1/15 patients, 6.7%), which was bladder perforation during placement.

All six complications required explantation and/or placement of a Foley catheter with local anesthesia. Therefore, these complications were graded as Clavien–Dindo Grade IIIa events. One prior radiated patient required immediate revision/reimplantation due to device malfunction. The device was removed after filling and demonstrated a small hole in the balloon via extravasation of contrast observed under direct vision with fluoroscopic evaluation. Two patients had complete device explantation. One patient required device explantation because of an unrelated emergency room admission that resulted in traumatic Foley catheterization and exposure of the device into the urethra. This patient opted for removal of the device and placement of an AUS. The other patient requested complete device explantation after unilateral balloon erosion into the bladder. No revisions were necessary for any patients outside of complications described.

## Discussion

This study assessed the safety, efficacy, and patient-reported outcomes of the ProACT device for men with PPUI. Across all the patients evaluated, the ProACT device demonstrated improvements in continence and quality of life metrics, with most patients reporting meaningful reductions in PPD and overall satisfaction with the device. However, when stratified by prior radiation status, the efficacy of the device, patient satisfaction, and complications varied.

While there was no significant difference in the reduction of PPD between the radiated and non-radiated groups, non-radiated patients were significantly more likely to achieve social dryness. The lack of statistical significance may be due to the small sample size and variability within the groups. This distinction highlights the challenges in the management of SUI in radiated patients. Despite the limitations of ProACT in radiated patients, there was no statistically significant difference between the radiated and non-radiated patients in recommending the ProACT device to others. This indicates that there could still be potential benefits to offering ProACT in radiated patients, especially when other treatments have failed.

This study population represents the surgeon’s first 21 DBACT patients. The average procedure time was 61.5 min, with the fastest case taking only 36 min, which more closely reflects the current average operating time after this physician has tripled this volume of procedures in non-radiated patients. This data set included cases which included resident physicians who operated alongside the primary surgeon at a teaching hospital, which likely accounts for the increased average operating time. As a surgeon becomes more familiar with the DBACT procedure and completes more cases, operating time is expected to decrease.

Patients who underwent RT generally required more adjustments but needed a lower total balloon volume. This was likely to accommodate the need for slow titration, preventing damage to fragile radiated surrounding tissues and allowing gradual urethral coaptation. Despite these precautions, radiated patients still required more revisions (3 of 6 radiated patients versus 1 of 15 non-radiated patients) compared to their non-radiated counterparts.

Of the study’s six total complications, five occurred in patients with prior radiation. This difference in complication rates was significant. However, two complications were potentially unrelated to the patient’s radiation status. One patient had a balloon that developed a small hole post-implantation, necessitating removal and reimplantation. Another previously radiated patient had a traumatic Foley catheterization during an ER admission for a non-urologic-related emergency, resulting in total device removal per the patient’s request. At their preoperative appointment, patients are instructed to wear a medical bracelet to alert medical professionals of the DBACT device to avoid such complications. The three complications in the radiated patients unrelated to device failure or traumatic catheterization were due to device erosion into the urethra, device erosion into the bladder, and intraoperative bladder perforation.

The worst complication among our patients was device erosion. This can occur with the AUS; however, the AUS requires intraoperative removal. In contrast, the removal of DBACT device is less invasive and can be done in the clinic. Studies investigating erosion rates after AUS implantation found rates of erosion to be 3.6–4.9% in non-radiated patients and 13–18% in radiated patients [11–13]. One 1277 patient multicenter study found rates of infection with the AUS to be 5.8% in non-radiated patients and 9.5% in radiated patients [14].

Device erosion into the bladder can be due to poor initial positioning or excessive initial inflation. Device removal usually resolves all long-term complications. Patients may undergo device reimplantation or receive more invasive treatment at a later date [15]. While complication rates appear to be lower for non-radiated patients in this study population, more data is needed to determine significance. Currently, further research is needed to assess the safety and efficacy of DBACT in patients with a history of pelvic radiation. To date, no patients have experienced chronic sequelae from the DBACT procedure, including fistula formation, recurrent urinary tract infections, chronic retention, or notably, erosion beyond the urethra into another non-urologic structure such as the rectum.

The primary limitation of this study is the sample size of 21 patients. The small sample size limits the power of any statistical analysis and the external validity of the findings. Large standard deviations observed for several parameters reflect substantial interpatient variability, limiting statistical power and suggesting heterogeneity in disease severity and treatment response. Low power also increases the risk of type II error in subgroup analysis between radiated and non-radiated patients. The retrospective single-surgeon, single-center design experience inherently limits generalizability and increases the potential for selection bias. While inclusion criteria were defined to include men with persistent moderate-to-severe stress incontinence refractory to conservative therapy, future multicenter prospective studies are needed for validation. The study, being non-randomized and without a control group, also increases the risks of selection bias. Candidates for ProACT implantation may have been healthier or less severe than patients offered other treatments such as AUS. Another limitation of this study is the relatively short follow-up period, with most patients evaluated for < 2 years, which may underestimate late complications or overstate the durability of continence and quality-of-life improvements. The use of survey data to measure quality of life outcomes also predisposes the study to recall bias and reporting bias, which could inflate positive outcomes and mask dissatisfaction. Objective 24-h pad weight testing would have provided greater accuracy but was unavailable in routine clinical practice. This was partially mitigated by also using chart review to track PPD changes.

## Conclusion

In this single-center study, the DBACT device for urethral coaptation demonstrated efficacy in safely improving continence and enhancing the QoL of patients with PPUI, as evidenced by a reduction in daily pad usage and improvements in IQoL scores. In the small population of radiated patients, there was a significantly higher complication rate and a trend toward less improvement compared to non-radiated patients. Despite these differences, both groups showed comparable reductions in daily pad usage. Overall, these findings suggest that the DBACT is a viable option as a minimally invasive and adjustable treatment for PPUI. Future studies with larger populations and control groups are needed to further characterize the durability, safety, and comparative efficacy of the DBACT in diverse patient populations.

## Supplementary Information

Below is the link to the electronic supplementary material.

Supplementary file1 (PNG 186 KB)

Supplementary file2 (DOCX 16 KB)

## Data Availability

The data supporting the findings of this study are available from the corresponding author upon reasonable request.

## Declarations

### Clinical trial registration

This study was not part of a clinical trial and did not require clinical trial registration. The ProACT device was used as part of standard clinical care rather than as an investigational intervention.

### Permission to reproduce material from other sources

All necessary permissions to reproduce previously published material have been obtained.
