# Supplementary figures and images for: Adjustable dual-balloon therapy: quality of life after prostate incontinence treatment
Source: Int Urol Nephrol. 2025 Dec 4;58(6):1971–8. doi: 10.1007/s11255-025-04945-w (PMC13194227; doi:10.1007/s11255-025-04945-w)

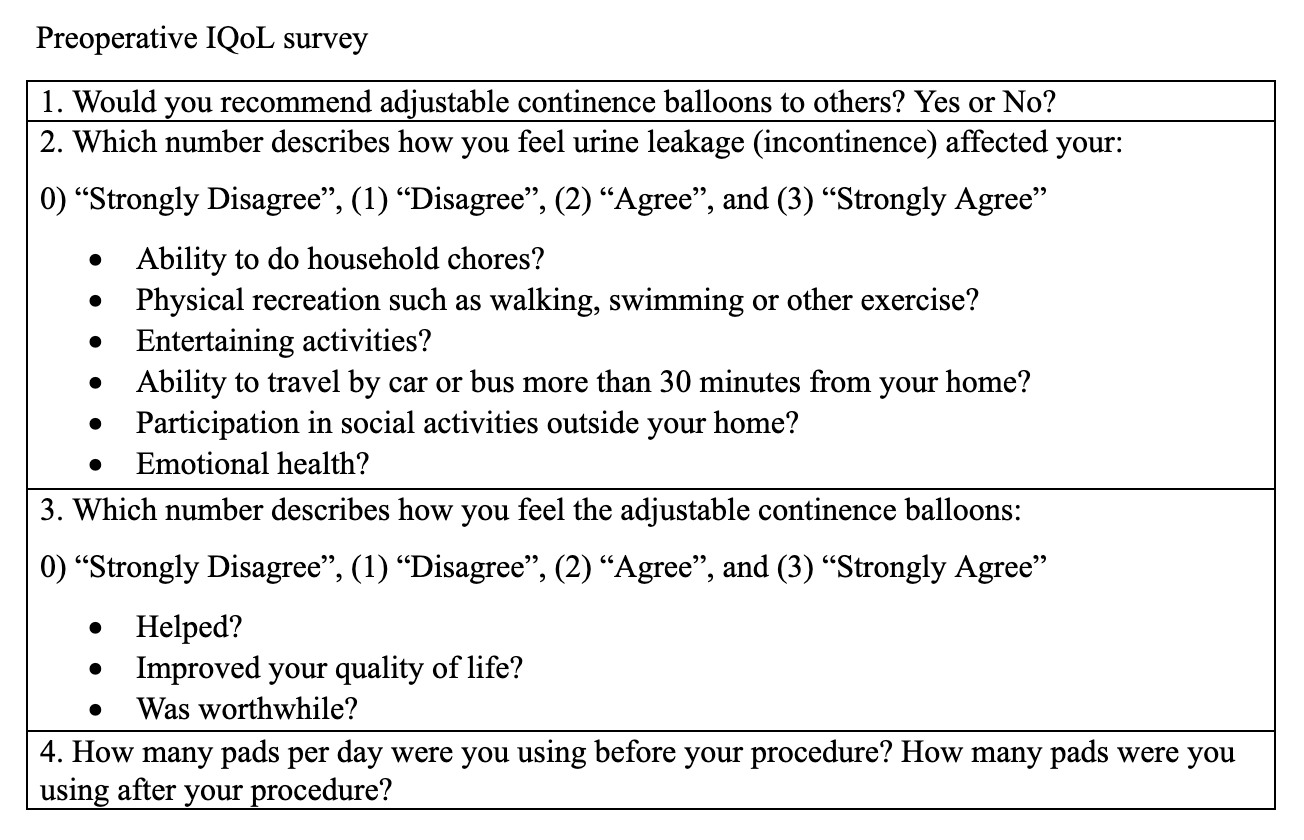

Supplement: Supplementary file 1 — Supplementary file1 (PNG 186 KB) [file 11255_2025_4945_MOESM1_ESM.png]
