# Supplementary material for: Adjustable dual-balloon therapy: quality of life after prostate incontinence treatment
Source: Int Urol Nephrol. 2025 Dec 4;58(6):1971–8. doi: 10.1007/s11255-025-04945-w (PMC13194227; doi:10.1007/s11255-025-04945-w)
Supplement: Supplementary file 2 — Supplementary file2 (DOCX 16 KB) [file 11255_2025_4945_MOESM2_ESM.docx]

**Supplementary Tables:**

Operative characteristics for initial implantation.

|  | Total (n = 21) | Radiated (n = 6) | Non-Radiated (n = 15) |
| --- | --- | --- | --- |
| Operating time, min | 61.14 (SD=17.23) | 64.67 (SD=15.42) | 59.73 (SD=18.21) |
| EBL, mL | 3.20 (SD=2.20) | 2.67 (SD=2.07) | 3.40 (SD=2.29) |
| Number of adjustments | 3.33 (SD=2.24) | 4.00 (SD=3.22) | 3.07 (SD=1.79) |
| Initial balloon volume, mL  Left  Right | 1.43 (SD=0.18)  1.40 (SD=0.26) | 1.33 (SD=0.26)  1.25 (SD=0.42) | 1.47 (SD=0.13)  1.47 (SD=0.13) |
| Final balloon volume, mL  Left  Right | 3.95 (SD=2.07)  4.02 (SD=2.12) | 3.50 (SD=2.02)  3.50 (SD=1.64) | 4.13 (SD=2.13)  4.23 (SD=2.30) |

Complications rates for all operations.

|  | Total (n = 6) | Radiated (n = 5) | Non-Radiated (n = 1) |
| --- | --- | --- | --- |
| Complications (Total)  Intraoperative  Postoperative | 6 (28.6%)  1 (4.7%)  5 (23.8%) | 5 (83.3%)  0 (0%)  5 (83.3%) | 1 (6.7%)  1 (6.7%)  0 (0%) |
| DBACT Explanted (Total)  Total removal  Removed and replaced (revision) | 6 (28.6%)  2 (9.5%)  4 (19.0%) | 5 (83.3%)  2 (33.3%)  3(50.0%) | 1 (6.7%)  0 (0%)  1 (6.7%) |
